# Indications and contraindications to platelet‐rich plasma injections in musculoskeletal diseases in case of infectious, oncological and haematological comorbidities: A 2025 formal consensus from the GRIIP (International Research Group on Platelet Injections)

**DOI:** 10.1002/ksa.12682

**Published:** 2025-04-22

**Authors:** Florent Eymard, Karine Louati, Éric Noel, Redouane Abouqal, Philippe Adam, Fadoua Allali, Gabriel Antherieu, Jo Caers, Fabrice Cognasse, Hervé Collado, Christelle Darrieutort‐Laffite, Corinne Frère, Alain Frey, Mathilde Gavillet, Vincent Gremeaux, Mael Heiblig, Guy Jerusalem, Charlotte Joly, Jean‐François Kaux, Martin Lamontagne, Mathieu Leclerc, Philippe Léonard, Raphaël Lepeule, Daniel Lopez‐Trabada‐Ataz, Jérémy Magalon, Fabrice Michel, Paul Ornetti, Cécile Oury, Elvire Pons‐Tostivint, Fernando Real, Ghislaine Robert, Mikel Sanchez, Alain Silvestre, Hervé Bard

**Affiliations:** ^1^ Department of Rheumatology, Créteil AP‐HP Henri Mondor Hospital Créteil France; ^2^ Department of Rheumatology AP‐HP Saint Antoine Hospital Paris France; ^3^ Ransay Santé, Jean Mermoz Private Hospital, Santy Orthopedic Center Lyon France; ^4^ Department of Emergency Medical Services Ibn Sina Hospital Rabat Morocco; ^5^ Department of Public Health, Medical School and Pharmacy, Laboratory of Biostatistics, Clinical and Epidemiological Research University Mohammed V Rabat Morocco; ^6^ Imaging Department Médipôle Garonne Clinic Toulouse France; ^7^ Department of Rheumatology El Ayachi Hospital Salé Morocco; ^8^ Department of Hematology Lyon University Hospital Lyon France; ^9^ Department of Clinical Hematology Liège University Hospital Liège Belgium; ^10^ French Blood Establishment Auvergne‐Rhône‐Alpes Saint Étienne France; ^11^ INSERM, U 1059 SAINBIOSE, Université Jean Monnet, Mines Saint‐Étienne Saint‐Etienne France; ^12^ Sports Health Medical Institute Marseille France; ^13^ Department of Rheumatology Nantes University Hospital Nantes France; ^14^ Department of Biological Hematology AP‐HP Pitié‐Salpêtrière Hospital Paris France; ^15^ Department of Sports Medicine Saint Germain en Laye, Saint Germain Hospital Poissy France; ^16^ Department of Hematology Lausanne University Hospital Lausanne Switzerland; ^17^ Division of Physical Medicine and Rehabilitation, Swiss Olympic Medical Center Lausanne University Hospital, Sport Medicine Unit Lausanne Switzerland; ^18^ Department of Oncology Liège University Hospital and Liege University Liège Belgium; ^19^ Department of Oncology AP‐HP Henri Mondor Hospital Créteil France; ^20^ Department of Physical and Rehabilitation Medicine Liège University Hospital Liège Belgium; ^21^ Department of Physical and Rehabilitation Medicine Montreal University Hospital Montreal Canada; ^22^ Department of Clinical Hematology AP‐HP Henri Mondor Hospital Créteil France; ^23^ Department of Infectious and Tropical Diseases Liège University Hospital Liège Belgium; ^24^ Department of Microbiology AP‐HP Henri Mondor Hospital Créteil France; ^25^ Department of Oncology AP‐HP Saint Antoine Hospital Paris France; ^26^ AP‐HM Hôpital De La Conception, Cell Therapy Laboratory Marseille France; ^27^ Department of Physical Medicine and Rehabilitation Jean‐Minjoz University Hospital Besançon France; ^28^ Department of Rheumatology Dijon University Hospital, Plateforme d'investigation Technologique CIC‐P INSERM 1432 Dijon France; ^29^ Cardiology Laboratory, GIGA Research Institute University of Liège Liège Belgium; ^30^ Department of Medical Oncology Nantes University Hospital Nantes France; ^31^ CNRS, Inserm, CHU Lille, Institut Pasteur de Lille, U1019–UMR 9017–CIIL, Center for Infection and Immunity of Lille L'Université de Lille Lille France; ^32^ Medical Office Redmond Washington USA; ^33^ Vithas Vitoria Hospital Arthroscopic Surgery Unit Vitoria Spain; ^34^ Imaging Department Osteoarthritis Center Bordeaux France; ^35^ Medical Office Vaudoyer Paris France

**Keywords:** contraindications, formal consensus, musculoskeletal diseases, PRP, recommendations

## Abstract

**Purpose:**

Platelet‐rich plasma (PRP) could be a vector for certain diseases, and its composition may vary by pathologic condition. The main comorbidities that could affect PRP composition are infectious, oncologic and haematologic. In addition to potential alteration of clinical response, these pathologies could have a significant impact on the local tolerance of PRP as well as a risk of disease dissemination to the injection site. To date, there are few specific recommendations related to these comorbidities to guide clinicians. Therefore, the International Research Group on Platelet Injections (GRIIP) supported a consensus project to develop these recommendations.

**Methods:**

Following the ‘recommendations by formal consensus’ methodology, a steering committee performed a literature review and drafted an initial set of recommendations. They were evaluated by an international rating group (15 specialists in musculoskeletal [MSK] diseases, five haematologists, four oncologists, three infectiologists and four scientists specialising in platelet physiology). From this rating, the first set of recommendations was discussed in a plenary meeting and then modified by the steering committee. Finally, four overarching principles and 23 recommendations were re‐evaluated by the rating group. Recommendations were classified as appropriate or not, with strong or relative agreement, or uncertain.

**Results:**

From the 23 recommendations, 10 concerned infectious diseases (viral and bacterial infections; dialysis; immunosuppressive drugs; dental care…), five oncologic diseases (local tumour; cured, active or in remission cancer…) and eight haematologic diseases (cytopenia; cured, active or stabilised cured hemopathy; monoclonal gammopathy…). All were considered appropriate by the experts (median = 9; range = 8–9), with strong or relative agreement. Due to the paucity of literature data, the recommendations are mainly based on expert opinion (Grade D).

**Conclusion:**

This consensus project provides four overarching principles and 23 recommendations related to contraindications of PRP injections in case of infectious, oncologic or hematologic diseases, validated by an international expert group.

**Level of Evidence:**

Level I.

**Abbreviations**
GRIIP: Groupe de Recherche International sur les Injections de Plaquettes (International Research Group on Platelet Injections)
HBV: hepatitis B Virus
HCV: hepatitis C Virus
HIV: human immunodeficiency virus
MGUS: monoclonal gammopathy of undetermined significance
MSK: musculoskeletal
NSAIDs: nonsteroidal anti‐inflammatory drugs
OA: osteoarthritis
OP: overarching principles
PRP: platelet‐rich plasma
R: recommendation

## INTRODUCTION

Autologous platelet‐rich plasma (PRP) injections are being used exponentially in musculoskeletal (MSK) diseases, most notably knee osteoarthritis (OA), but also in other OA sites and various tendinopathies (e.g., rotator cuff, gluteal or patellar tendons) [34] in case of failure of non pharmacological treatments. Their success is due to their efficacy, which seems at least equivalent to and often superior to that of other injectable products and probably longer‐lasting, both in OA [12, 22] and in certain tendinopathies [24, 29], and to their excellent tolerance profile. In fact, PRP injections have few adverse effects, apart from the fairly common occurrence of a transient increase in pain, sometimes accompanied by mild local inflammation at the injection site [12].

However, because of its complex composition, mainly plasma and platelets but also other cell types (white blood cells and red blood cells), PRP can be a vector for certain diseases, transporting pathogens or metastatic cells to the injection site. In addition, a given comorbidity can influence platelet metabolism, in particular its secretory profile, for example, by promoting the release of inflammatory and/or catabolic factors. These two phenomena, sometimes occurring simultaneously, could have a significant impact on the local tolerability profile of PRP, with increased pain or local inflammatory responses, as well as a risk of spreading an associated disease to the injection site. The main co‐morbidities encountered in current practice that may be 'transported' by PRP and/or have an impact on the platelet phenotype are, of course, infectious pathologies, either acute or chronic, bacterial or viral, local or distant neoplastic pathologies and hematologic pathologies.

To date, the recommendations for good practice issued by various professional societies have only imprecisely addressed certain associated diseases, such as cancers and infections, which are considered contraindications because of general and poorly justified precautionary principles [21, 33]. However, there are no specific recommendations based on precise clinical situations (e.g., active cancer or remission; presence of metastasis or not; viral or bacterial infection) to better guide clinicians. This leads to heterogeneous practices among clinicians in these increasingly common clinical situations.

The aim of this consensus project promoted by the International Research Group on Platelet Injections (Groupe de Recherche International sur les Injections de Plaquettes [GRIIP]), which is an international group of experts in the field of PRP injections, was to provide precise recommendations for PRP injections in patients with infectious, oncologic or hematologic diseases.

## MATERIALS AND METHODS

### Methodology of the recommendations

We followed a methodology validated by the French Health Authority called ‘recommendations by formal consensus’ [28]. This methodology is suitable for developing recommendations for which there is lack of scientific literature and which therefore rely mainly on expert opinion. It is also internationally recognised [7, 8] and has been used on several occasions, most recently for the development of recommendations on orthobiologic treatments for OA [21, 33]. The members of the steering group were validated by the GRIIP scientific committee while the members of the rating group were proposed by the steering group to the GRIIP Scientific Committee, which also validated them.

### Steering group

The steering group including three GRIIP members (FE, KL and EN) met a first time to identify and discuss the clinical situations that warranted inclusion in these recommendations. Each member of the steering group was responsible for a medical field (infectiology, oncology and haematology). A targeted literature search was then performed by the members of the steering group between February 2021 and May 2021 on Medline (https://pubmed.ncbi.nlm.nih.gov/), according to keywords relevant to each clinical situation. The abstracts of all references were assessed and the full text of all relevant articles was obtained. Only papers published in English were considered, with no restriction on publication date or level of evidence. Sixty‐five articles were selected. Each member then drafted an initial set of recommendations for their medical field based on the literature found. Then, in a second meeting attended by the three members of the steering group, literature data were presented, and the different principles and recommendations were discussed and modified until all three members of the group agreed on each statement. The members of the steering group did not participate in rating the recommendations.

### Rating group

We then gathered 31 experts from France, Switzerland, Spain, Belgium, Morocco, Canada and the United States, including 15 specialists in MSK disorders (sports physicians, rheumatologists, physical and rehabilitation medicine physicians, radiologists) who perform PRP injections, 12 clinicians with expertise in the pathologies covered by the recommendations (five haematologists, four oncologists and three infectious disease specialists) and four scientists specialising in platelet physiology and its involvement in various pathologies.

### Rating of recommendations

An initial list of seven overarching principles and 21 recommendations, together with the literature review, was presented to the 31 experts of the rating group at a first meeting. In addition, the presentation support, which summarised the literature data, was sent to all of them. Following this meeting, because of their more transversal vision, the 15 experts specialising in MSK diseases and the four scientists rated all 28 items, and the five haematologists, four oncologists and three infectious disease specialists rated only those items related to their speciality, as well as the seven overarching principles.

The rating was based on a simple numerical scale from 1 to 9, a score of 1 indicating that the expert considered the proposal to be ‘totally inappropriate’ (or not indicated or not acceptable), a score of 9 indicating that the expert considered the proposal to be 'totally appropriate' (or indicated or acceptable), scores of 2–8 reflecting possible intermediate situations, and a score of 5 corresponding to the expert's indecision. The scores for all experts were then pooled and the median and range were calculated. No rating was excluded at the end of this first round of scoring. A proposal was considered appropriate if the median was ≥7 and there was agreement among members of the rating group (strong agreement if the distribution of ratings was 7–9 and relative agreement if 5–9). The proposal was considered inappropriate if the median was ≤3.5 and there was agreement among members of the rating group (strong agreement if the median was ≤3 and the distribution of ratings was 1–3, relative agreement if the median was ≤3.5 and the distribution was 1–5). The proposal was considered uncertain if the median was from 4 to 6.5 (indecision) or in all other situations not described above (lack of consensus).

Following this first assessment, a second meeting including the steering group and raters was held to review the experts' ratings and arguments for each of the principles and recommendations, with the exception of those that were considered appropriate with strong agreement from the first round and were therefore retained without change. All other recommendations were modified in substance or form or were withdrawn by the steering group, taking into account the arguments put forward by the experts at the time of their initial evaluation and/or during this second meeting. Thus, three general principles were withdrawn. In addition, following discussions with the raters, two recommendations have been split into two to improve clarity.

Four overarching principles and 23 recommendations were then retained and were subject to a second scoring. In accordance with the formal consensus methodology, because of more than 15 experts included, two extreme ratings were excluded for each recommendation with a median ≥7 or ≤3.5 to assess the level of agreement. Thus, strong agreement was obtained if all responses were 7–9 or 1–3, excluding two responses, and relative agreement if all responses were 5–9 or 1–5, excluding two responses.

For each overarching principle and recommendation, we assigned a grade based on the level of evidence from literature data and expert opinion. The level of evidence depending of study design was defined as followed: [10] 1A, Systematic review (with homogeneity) of RCTs; 1B, Individual RCT (with narrow confidence intervals); 2A, Systematic review (with homogeneity) of cohort studies; 2B, Individual Cohort study (including low quality RCT, e.g., <80% follow‐up); 3A, Systematic review (with homogeneity) of case–control studies; 3B, Individual Case–control study; 4, Case series (and poor quality cohort and case–control study); 5, Expert opinion without explicit critical appraisal or based on physiology bench research or ‘first principles’. The grade of recommendation was then defined as followed: [33] A: At least three Level of evidence 1 papers; B: At least three Level of evidence 2 papers; C: Cohort series/comparative studies, with concordant conclusions or high‐level studies but with contradictory or nonconclusive results; D: Expert opinion of the group given the very poor or absent literature.

### Review committee

The final stage in the development of the recommendations was to submit them to a review committee of 31 GRIIP members who specialise in MSK diseases and perform PRP injections (16 sports physicians, 8 rheumatologists, 6 physical and rehabilitation medicine physicians and 1 radiologist). They assessed the readability of each principle and recommendation and could comment them. Their opinion was ‘advisory’ and did not allow the recommendations to be revised, but their arguments could be added to those of the raters.

## RESULTS

Four overarching principles and 23 recommendations were assessed by the 31 experts in the second scoring. These overarching principles are a general reminder of good clinical practice for PRP injections and serve to introduce the more specific recommendations. Of the 23 recommendations, 10 were related to infectious diseases, five to oncology and eight to haematological diseases. All were considered appropriate by the experts (median = 9; range = 8–9), with strong agreement (four overarching principles and 21 recommendations) or relative agreement (two recommendations). Due to a lack of evidence in the literature, the recommendations are based mainly on clinical expertise. As a result, most recommendations are Grade D, with the exception of recommendations 1, 5, 7, 8, 9 and 13, which are Grade C. The full version of arguments for each overarching principle and recommendation is available in Supporting Information: File 1.

**Overarching principle 1: The indication for PRP injection for joint or tendon disease must be established by a specialist in musculoskeletal diseases** (Table 1).

Grade D

**Table 1:** Overarching principles.

		All ratings			After excluding 2 extreme ratings					
		No. of ratings	Median	Range	No. of ratings	Median	Range	Judgement and degree of agreement	Grade	References
OP1	The indication of PRP injection for joint or tendon disease must be established by a specialist in musculoskeletal diseases.	31	9	9–9	29	9	9–9	Appropriate with strong agreement	Grade D	[16, 31, 40, 51]
OP2	Each injection of PRP must be preceded by an assessment of the benefit–risk balance, taking into account the indication for injection and the characteristics of the lesion as well as the patient's history, comorbidities and treatments while systematically considering injectable therapeutic alternatives (corticosteroids, hyaluronic acid, etc.).	31	9	7–9	29	9	8–9	Appropriate with strong agreement	Grade D	[17, 39, 48]
OP3	In the presence of co‐morbidities that may compromise platelet function or be transmitted by PRP, this treatment should be considered only in the absence of efficacious alternative therapeutic options or following their failure.	31	9	7–9	29	9	7–9	Appropriate with strong agreement	Grade D	[35, 37]
OP4	Patients must receive clear, personalised information about the potential benefits and risks of PRP injection, taking into account the medical history and comorbidities, and the patient's informed consent must be recorded in the medical file.	31	9	8–9	29	9	9–9	Appropriate with strong agreement	Grade D	[39, 50]

Abbreviation: PRP, platelet‐rich plasma.

**Appropriate with strong agreement**

Number of ratings: 29

Median = 9; range = 9–9

**Overarching principle 2: Each PRP injection must be preceded by an assessment of the benefit–risk balance, taking into account the indication for injection and the characteristics of the lesion as well as the patient's history, comorbidities and treatments while systematically considering injectable therapeutic alternatives (corticosteroids, hyaluronic acid, etc.)**.

Grade D

**Appropriate with strong agreement**

Number of ratings: 29

Median = 9; range = 8–9

**Overarching principle 3: In the presence of co‐morbidities that may compromise platelet function or be transmitted by PRP, this treatment should be considered only in the absence of efficacious alternative therapeutic options or following their failure.**

Grade D

**Appropriate with strong agreement**

Number of ratings: 29

Median = 9; range = 7–9

**Overarching principle 4: Patients must receive clear, personalised information about the potential benefits and risks of PRP injection, taking into account the medical history and comorbidities, and the patient's informed consent must be recorded in the medical file.**

Grade D

**Appropriate with strong agreement**

Number of ratings: 29

Median = 9; range = 9–9

**Recommendation 1: In patients with HIV infection, PRP injection may be performed if the viral load is undetectable and the CD4 count is >350/mm^3^** (Table 2).

Grade C

**Table 2:** Recommendations for infectious diseases.

		All ratings			After exclusion of 2 extreme ratings					
		No. of ratings	Median	Range	No. of ratings	Median	Range	Judgement and degree of agreement	Grade	References
R1	In patients with HIV infection, PRP injection may be performed if the viral load is undetectable and the CD4 count is >350/mm^3^.	22	8	5–9	20	8	7–9	Appropriate with strong agreement	Grade C	[1, 5, 30, 44, 52]
R2	In patients with hepatitis B virus infection, PRP injection may be performed if the viral load is undetectable.	22	8	5–9	20	8.5	7–9	Appropriate with strong agreement	Grade D	[13]
R3	In patients with hepatitis C virus infection, PRP injection should be performed only after antiviral treatment has been completed.	22	9	5–9	20	9	7–9	Appropriate with strong agreement	Grade D	[3, 15, 42]
R4	In patients with an acute viral infectious syndrome, PRP injection may be performed if the patient has no general symptoms (fever, chills, etc.) or signs of bacterial infection and if the viral syndrome is improving.	22	8	2–9	20	8	7–9	Appropriate with strong agreement	Grade D	[11]
R5	In patients with a bacterial infection requiring antibiotic therapy of less than 3 months, PRP injection may only be performed once the treatment has been completed.	22	9	7–9	20	9	8‐9	Appropriate with strong agreement	Grade C	[14, 49, 52]
R6	In patients with a controlled bacterial infection requiring antibiotic therapy for more than 3 months, a PRP injection may be performed only after infectiologist agreement.	22	9	6–9	20	9	8‐9	Appropriate with strong agreement	Grade D	[4, 9, 25, 26]
R7	In patients with chronic renal failure requiring dialysis, PRP injection may be considered, but the patient should be carefully monitored for signs of bacteremia and the injection must be avoided on the day of dialysis.	22	8	3–9	20	8	5–9	Appropriate with relative agreement	Grade C	[18, 27, 46]
R8	In patients with prolonged and stabilised prescription of immunosuppressive agents, PRP injection may be performed, but particular attention must be paid to the presence of concomitant infection.	22	8	5–9	20	8.5	7‐9	Appropriate with strong agreement	Grade C	[6, 23]
R9	In patients with dental infection or invasive oral procedures, PRP injection should not be performed until the completion of treatment and healing.	22	9	8–9	20	9	9–9	Appropriate with strong agreement	Grade C	[38, 47]
R10	In asymptomatic patients with no known infectious pathology, no additional tests for infection are needed.	22	9	5–9	20	9	9–9	Appropriate with strong agreement	Grade D	

Abbreviations: HIV, human immunodeficiency virus; PRP, platelet‐rich plasma.

**Appropriate with strong agreement**

Number of ratings: 20

Median = 8; range = 7–9

**Recommendation 2: In patients with hepatitis B virus infection, PRP injection may be performed if the viral load is undetectable.**

Grade D

**Appropriate with strong agreement**

Number of ratings: 20

Median = 8.5; range = 7–9

**Recommendation 3: In patients with hepatitis C virus infection, PRP injection should be performed only after antiviral treatment has been completed.**

Grade D

**Appropriate with strong agreement**

Number of ratings: 20

Median = 9; range = 7–9

**Recommendation 4: In patients with an acute viral infectious syndrome, PRP injection may be performed if the patient has no general symptoms (fever, chills, etc.) or signs of bacterial infection and if the viral syndrome is improving.**

Grade D

**Appropriate with strong agreement**

Number of ratings: 20

Median = 8; range = 7–9

**Recommendation 5: In patients with a bacterial infection requiring antibiotic therapy of less than 3 months, PRP injection may only be performed once the treatment has been completed.**

Grade C

**Appropriate with strong agreement**

Number of ratings: 20

Median = 9; range = 8–9

**Recommendation 6: In patients with a controlled bacterial infection requiring antibiotic therapy for more than 3 months, PRP injection may be performed only after infectiologist agreement.**

Grade D

**Appropriate with strong agreement**

Number of ratings: 20

Median = 9; range = 8–9

**Recommendation 7: In patients with chronic renal failure requiring dialysis, PRP injection may be considered, but the patient should be carefully monitored for signs of bacteremia and the injection must be avoided on the day of dialysis.**

Grade C

**Appropriate with relative agreement**

Number of ratings: 20

Median = 8; range = 5–9

**Recommendation 8: In patients with prolonged and stabilised prescription of immunosuppressive agents, PRP injection may be performed, but particular attention must be paid to the presence of concomitant infection.**

Grade C

**Appropriate with strong agreement**

Number of ratings: 20

Median = 8.5; range = 7–9

**Recommendation 9: In patients with dental infection or invasive oral procedures, PRP injection should not be performed until the completion of treatment and healing.**

Grade C

**Appropriate with strong agreement**

Number of ratings: 20

Median = 9; range = 9–9

**Recommendation 10: In asymptomatic patients with no known infectious pathology, no additional tests for infection are needed.**

Grade D

**Appropriate with strong agreement**

Number of ratings: 20

Median = 9; range = 9–9

**Recommendation 11: PRP should not be injected in the vicinity of benign or malignant tumours (bone, synovial or soft tissue) or metaplasia (such as primary synovial chondromatosis)** (Table 3).

Grade D

**Table 3:** Recommendations for oncologic diseases.

		All ratings			After exclusion of 2 extreme ratings					
		No. of ratings	Median	Range	No. of ratings	Median	Range	Judgement and degree of agreement	Grade	References
R11	PRP should not be injected in the vicinity of benign or malignant tumours (bone, synovial or soft tissue) or metaplasia (such as primary synovial chondromatosis).	23	9	7–9	21	9	8–9	Appropriate with strong agreement	Grade D	[2, 35, 36]
R12	In patients with solid cancers undergoing diagnosis or considered active, PRP injection should not be performed, except in exceptional situations to be discussed with the oncologist.	23	9	8–9	21	9	9–9	Appropriate with strong agreement	Grade D	[32, 35]
R13	In patients with non‐metastatic solid cancer considered in remission by the oncologist after the end of treatment, PRP injection may be performed.	23	9	1–9	21	9	7–9	Appropriate with strong agreement	Grade C	[19, 20]
R14	In patients with metastatic solid cancer under treatment or not and considered in remission, PRP injection may be performed only after oncologist agreement.	23	9	7–9	21	9	8–9	Appropriate with strong agreement	Grade D	
R15	In patients with a history of solid cancer that the oncologist considers cured, PRP injection may be performed.	23	9	1–9	21	9	7–9	Appropriate with strong agreement	Grade D	

Abbreviation: PRP, platelet‐rich plasma.

**Appropriate with strong agreement**

Number of ratings: 21

Median = 9; range = 8–9

**Recommendation 12: In patients with solid cancers undergoing diagnosis or considered active, PRP injection should not be performed, except in exceptional situations to be discussed with the oncologist.**

Grade D

**Appropriate with strong agreement**

Number of ratings: 21

Median = 9; range = 9–9

**Recommendation 13: In patients with non‐metastatic solid cancer considered in remission by the oncologist after the end of treatment, PRP injection may be performed.**

Grade C

**Appropriate with strong agreement**

Number of ratings: 21

Median = 9; range = 7–9

**Recommendation 14: In patients with metastatic solid cancer under treatment or not and considered in remission, PRP injection may be performed only after oncologist agreement.**

Grade D

**Appropriate with strong agreement**

Number of ratings: 21

Median = 9; range = 8–9

**Recommendation 15: In patients with a history of solid cancer that the oncologist considers cured, PRP injection may be performed.**

Grade D

**Appropriate with strong agreement**

Number of ratings: 21

Median = 9; range = 7–9

**Recommendation 16: A significant abnormality in the blood count should be investigated before PRP injection** (Table 4).

Grade D

**Appropriate with strong agreement**

Number of ratings: 22

**Table 4:** Recommendations for hematologic diseases.

		All ratings			After exclusion of 2 extreme ratings					
		No. of ratings	Median	Range	No. of ratings	Median	Range	Judgement and degree of agreement	Grade	References
R16	A significant abnormality in the blood count should be investigated before PRP injection.	24	9	7–9	22	9	9–9	Appropriate with strong agreement	Grade D	
R17	Previously investigated thrombocytopenia > 50,000/mm3, excluding hematologic malignancies, is not a contraindication to PRP injection.	24	8	3–9	22	8	7–9	Appropriate with strong agreement	Grade D	
R18	In patients with a hematologic malignancy undergoing diagnosis or not considered stabilised, PRP injection should not be performed, except in exceptional situations to be discussed with the haematologist.	24	9	1–9	22	9	8–9	Appropriate with strong agreement	Grade D	
R19	In patients with a hematologic malignancy considered in remission by the haematologist after the completion of treatment (including post‐transplant immunosuppressive therapy), PRP injection may be performed if there are no platelet count abnormalities.	24	8.5	7–9	22	9	7–9	Appropriate with strong agreement	Grade D	[41]
R20	In patients with stabilised chronic lymphoid hemopathy under treatment or not, PRP injection may be performed if there are no platelet count abnormalities.	24	8.5	6–9	22	9	8–9	Appropriate with strong agreement	Grade D	[41]
R21	In patients with a stabilised chronic myeloid hemopathy under treatment or not, PRP injection may be performed only after haematologist agreement.	24	9	6–9	22	9	8–9	Appropriate with strong agreement	Grade D	
R22	In patients with monoclonal gammopathy of undetermined significance, PRP injection may be performed.	24	9	5–9	22	9	6–9	Appropriate with relative agreement	Grade D	[43]
R23	In patients with a hematologic malignancy that the haematologist considers cured, PRP injection may be performed.	24	9	7–9	22	9	8–9	Appropriate with strong agreement	Grade D	

Abbreviation: PRP, platelet‐rich plasma.

Median = 9; range = 9–9

**Recommendation 17: Previously investigated thrombocytopenia > 50,000/mm^3^, excluding hematologic malignancies, is not a contraindication to PRP injection.**

Grade D

**Appropriate with strong agreement**

Number of ratings: 22

Median = 8; range = 7–9

**Recommendation 18: In patients with a hematologic malignancy undergoing diagnosis, or not considered stabilised, PRP injection should not be performed, except in exceptional situations to be discussed with the haematologist.**

Grade D

**Appropriate with strong agreement**

Number of ratings: 22

Median = 9; range = 8–9

**Recommendation 19: In patients with a hematologic malignancy considered in remission by the haematologist after the completion of treatment (including post‐transplant immunosuppressive therapy), PRP injection may be performed if there are no platelet count abnormalities.**

Grade D

**Appropriate with strong agreement**

Number of ratings: 22

Median = 9; range = 7–9

**Recommendation 20: In patients with stabilised chronic lymphoid hemopathy under treatment or not, PRP injection may be performed if there are no platelet count abnormalities.**

Grade D

**Appropriate with strong agreement**

Number of ratings: 22

Median = 9; range = 8‐9

**Recommendation 21: In patients with a stabilised chronic myeloid hemopathy under treatment or not, PRP injection may be performed only after haematologist agreement.**

Grade D

**Appropriate with strong agreement**

Number of ratings: 22

Median = 9; range = 8‐9

**Recommendation 22: In patients with monoclonal gammopathy of undetermined significance, PRP injection may be performed.**

Grade D

**Appropriate with relative agreement**

Number of ratings: 22

Median = 9; range = 6–9

**Recommendation 23: In patients with a hematologic malignancy that the haematologist considers cured, PRP injection may be performed.**

Grade D

**Appropriate with strong agreement**

Number of ratings: 22

Median = 9; range = 8–9

## DISCUSSION

This consensus project provides the first recommendations to specifically address respective indications and contraindications to PRP injection for MSK diseases in patients with infectious, oncological or haematological comorbidities. Indeed, PRP may be a vector for these diseases, transporting pathogens or metastatic cells to the injection site, while these comorbidities may influence platelet metabolism by promoting the release of inflammatory and/or catabolic factors. The full rationale for each overarching principle and recommendation can be found in Supporting Information: File 1. In brief, the experts considered that chronic viral infections were not a contraindication to PRP injections as long as patients were being or had been treated and had a negative viral load with, in the case of HIV, an adequate immune response (CD4 > 350/mm^3^). On the other hand, acute bacterial infections need to be cured before PRP injections, so it is necessary to wait until the end of antibiotic treatment to avoid the risk of inoculating bacteria at the injection site. For chronic infections that require prolonged treatment, PRP injections can be performed after at least 3 months of antibiotics and when the infectious disease specialist has confirmed that the infection is under control. In addition, PRP injections must be used with great caution in patients with diseases or treatments (dialysis, immunosuppressive treatment) that predispose them to bacterial infections, while they are contraindicated in dental care due to the high risk of bacteraemia. With regard to oncological pathologies, the experts consider that PRP injections should not be performed in the vicinity of local benign or malignant tumours due to the risk of promoting their growth via the factors released by the platelets. Similarly, active cancer is a contraindication to PRP injections, whether localised or metastatic, mainly because of the risk of carrying metastatic cells to the injection site. On the other hand, a cured or in remission cancer does not constitute a contraindication to PRP injections if local, while it needs the agreement of the oncologist if initially metastatic.

The recommendations are broadly similar for haematological disorders, that is, there is no contraindication to PRP injections in cured or in remission hemopathies. However, for hemopathies stabilised with or without treatment, the experts suggested that a distinction be made between lymphoid and myeloid diseases, as the risk of thrombopathy is higher in myeloid hemopathies, justifying the need for haematologist consent before PRP injection. Finally, the experts considered that thrombocytopenia greater than 50,000/mm^3^ and MGUS are not contraindications for PRP injections.

Although the recommendations could be extended to other PRP indications (e.g., stomatology, dermatology, gynaecologist and plastic surgery), we limited them to MSK conditions because we did not involve experts in these other indications. Moreover, we focused on only infectious, oncologic and hematologic diseases, which are the most problematic in current clinical practice. However, the list of clinical situations related to the pathologies included in our recommendations is not exhaustive and other situations may also be addressed. In addition, other associated pathologies could merit similar recommendations, such as inflammatory and autoimmune diseases, for which platelet hyperactivation could induce adverse effects, in particular via the pro‐inflammatory phenotype of the injected platelets [45]. On the other hand, these recommendations address only the risks specific to PRP and not those related to the procedure, such as the risk of bleeding or local infectious inoculation. Similarly, they do not address the risk of reduced efficacy of PRP due to its composition, such as congenital or acquired thrombocytopenia or thrombopathy. Finally, we chose not to address potential interactions with other treatments (non‐steroidal anti‐inflammatory drugs [NSAIDs], antiaggregants, anticoagulants, local anaesthetics, etc.) because they are mainly involved in reduced efficacy of PRP (NSAIDs, antiaggregants, local anaesthetics) or in adverse effects more related to the injection than to the product (anticoagulants and antiaggregants). Furthermore, these interactions have already been the subject of recommendations [21, 33].

The main aim of this work was to provide precise recommendations in several clinical situations, based on the opinion of numerous experts in both PRP and MSK diseases or associated conditions, to avoid systematic recourse to the opinion of a colleague who is not necessarily familiar with PRP. However, as we could not address all clinical situations, given their diversity and complexity, we encourage physicians to question their direct colleagues if they are uncertain about a particular situation. Furthermore, the precautionary principle dictates that if there is the slightest doubt, PRP injection, which is never an emergency, should be avoided. Finally, as reiterated in overarching principle 3, the indication for PRP injection in the situations listed above must be carefully weighed and should only be administered in cases of non‐indication, contraindication or failure of therapeutic alternatives.

The main strengths of our recommendations are their methodological rigour, based on a validated and widely used methodology, and their multidisciplinary aspect. Indeed, 31 experts from several countries contributed to their rating, including 15 specialists in MSK conditions; 12 clinicians specialising in infectious, oncologic or hematologic diseases; and four scientists with extensive multidisciplinary expertise in platelet function. Moreover, the level of agreement was strong for most recommendations, which reinforces their credibility. Indeed, only two recommendations were judged ‘appropriate with relative agreement’.

Our recommendations have also certain limits. The first is the lack of high medical evidence given the absence of data in the literature to support most of the recommendations, but this was precisely the reason for developing these recommendations based on clinical expertise and using the most appropriate methodology in this context. Moreover, the safety analyses of the many published randomised controlled trials did not provide information because most did not include patients with such comorbidities but also because the insufficient number of patients and the short duration of follow‐up would not have allowed complications related to the comorbidities listed here to emerge. Only large real‐life registries with a high number of patients followed for a sufficiently long time would provide answers.

We also acknowledge a deviation from the initial methodology, as not all the experts rated all the recommendations. In fact, the experts in haematology, infectious diseases and oncology only rated those relating to their speciality. This was decided because they had limited knowledge and no clinical experience of PRP, and no expertise in other specialities. Only MSK experts and scientists specialising in platelet physiology rated all the recommendations. However, we believe that the number of experts who assessed each recommendation (at least 22), well above the minimum required by the original methodology (nine members), limited the risk that the rigour of the methodology would have been compromised. The overall homogeneity of the scores given to each recommendation is another factor suggesting that the consensus among the members of the evaluation group would not have been significantly affected if all members had rated.

In addition, although the experts were international, most were European and mainly French. However, practices may vary among continents and physicians' attitudes on the risk involved in the medical decision may differ. In addition, the diagnostic and therapeutic management and surveillance of the diseases listed in these recommendations vary among countries, especially depending on ease of access to the healthcare system, which may affect the benefit–risk balance that the experts used as a guide. The same limit can be applied to the review committee, which was pluralistic in terms of medical speciality, but mainly French. On the other hand, we chose to include as MSK experts, only physicians who practice PRP injection. Indeed, we felt that participants needed to be familiar with PRP injection in order to give an informed opinion. This decision may have led to some indication bias because such participants may be less concerned about certain co‐morbidities, given their reassuring personal experience in their own practice. Hence, we also included experts in these different co‐morbidities who do not practise PRP injection, to obtain an objective and unbiased opinion.

We believe that these recommendations could have a significant impact on clinical practice by guiding physicians on whether or not to perform a PRP injection depending on the presence of comorbidities. However, they could also influence the design of future clinical trials, which could use them to adapt the inclusion or exclusion criteria. Of course, it would be very interesting to conduct trials to validate these recommendations and thus strengthen the level of evidence, but this seems complex given the risk‐benefit balance for a treatment that remains mainly symptomatic.

## CONCLUSION

These four overarching principles and 23 recommendations are intended to guide clinicians in the use of PRP injections in patients with several specific comorbidities, defined in as much detail as possible, taking into account their variability, in order to be as close as possible to current practice. Indeed, depending on the stage and/or severity of a comorbidity, a different approach is warranted. Thus, a PRP injection will be contraindicated in a patient with an active cancer while it will be authorised in patient with a local cancer in remission or considered cured. Similarly, a viral infection won't be considered in the same way as a bacterial infection. These recommendations are the first step before obtaining more solid scientific data, which will probably only come from large international real‐life registries. Given the low level of evidence for these recommendations and the impossibility of addressing every possible clinical situation, we encourage physicians to maintain interaction with their colleagues specialised in the disease in question whenever they have the slightest doubt.

## AUTHOR CONTRIBUTIONS

Florent Eymard, Karine Louati and Éric Noel contributed to the design, conducted a comprehensive literature review and wrote the manuscript. All authors except Florent Eymard, Karine Louati and Éric Noel rated the recommendations. All participated substantially in revising the manuscript before submission.

## CONFLICT OF INTEREST STATEMENT

Florent Eymard declares grants from RegenLab and support for attending meetings from Fidia. Jérémy Magalon is a co‐founder of Remedex and received honoraria for educational support from Fidia Pharmaceuticals, Horiba, Arthrex, Horus Pharma and Macopharma. OP received honoraria for educational support from Fidia Pharmaceuticals and RegenLab. Cécile Oury is Research Director at Belgian Funds for Scientific Research (F.R.S.‐FNRS). Other authors have no conflicts of interest.

## ETHICS STATEMENT

None declared.

## Supporting information

Supporting information.

## Data Availability

The data that support the findings of this study are available from the corresponding author upon reasonable request.
