# Supplementary material for: Indications and contraindications to platelet‐rich plasma injections in musculoskeletal diseases in case of infectious, oncological and haematological comorbidities: A 2025 formal consensus from the GRIIP (International Research Group on Platelet Injections)
Source: Knee Surg Sports Traumatol Arthrosc. 2025 Apr 22;33(6):2293–306. doi: 10.1002/ksa.12682 (PMC12104799; doi:10.1002/ksa.12682)
Supplement: Supplementary file 1 — Supporting information. [file KSA-33-2293-s001.docx]

**Overarching principles (Table 1)**

**Overarching principle 1: The indication for PRP injection for joint or tendon disease must be established by a specialist in musculoskeletal diseases.**

Grade D

**Appropriate with strong agreement**

Number of ratings: 29

Median = 9; range = 9–9

The efficacy of injectable treatments for “mechanical” or “degenerative” MSK pathologies are widely used and include a limited number of therapeutics. However, their efficacy varies widely depending on the specific pathology or site being treated. Therefore, expertise in MSK diseases is required for using these treatments effectively, particularly for PRP injections, which have a number of potential indications, some that have never been scientifically proven. In addition, there is considerable variability in the types of tissue lesions, or clinical symptoms or radiological severity that may affect the suitability of a particular injectable treatment. For example, for tendinopathies, lesions may include tendinosis, partial and complete tear, enthesopathy or rupture [16, 32, 52]. In OA, the radiographic severity differs among patients, and the clinical presentation can also vary considerably in terms of pain, functional limitations and inflammatory pattern (synovitis and/or effusion) [41]. Some of these lesions or clinical presentation are considered to justify or contraindicate a particular treatment such as corticosteroids injections. Conversely, experts believe that a physician does not necessarily have to perform the PRP injections in order to prescribe them.

**Overarching principle 2: Each PRP injection must be preceded by an assessment of the benefit–risk balance, taking into account the indication for injection and the characteristics of the lesion as well as the patient's history, comorbidities and treatments while systematically considering injectable therapeutic alternatives (corticosteroids, hyaluronic acid, etc.).**

Grade D

**Appropriate with strong agreement**

Number of ratings: 29

Median = 9; range = 8–9

The benefit–risk ratio of PRP injections must be assessed as for any other treatment, taking into account its indication (OA, tendinopathy, etc.); the location, chronicity and severity of the lesion, which may influence the choice of treatment (see Overarching principle 1); and, of course, comorbidities and medical history, which may constitute potential contraindications or precautions for use, particularly those addressed in these recommendations. Furthermore, one must systematically consider the benefits of other injectable treatments compared to PRP in this specific indication [17, 49], also taking into account comorbidities that may affect their tolerability (e.g. diabetes and corticosteroids) [40].

**Overarching principle 3: In the presence of co-morbidities that may compromise platelet function or be transmitted by PRP, this treatment should be considered only in the absence of efficacious alternative therapeutic options or following their failure.**

Grade D

**Appropriate with strong agreement**

Number of ratings: 29

Median = 9; range = 7–9

This recommendation is complementary to the previous one and is intended to highlight the special characteristics of PRP, which is a "living" autologous product composed of the patient's plasma and cells (including, but not limited to platelets). Consequently, PRP has the potential to act as a vector for a number of diseases via the plasma and/or cells and could theoretically promote diffusion at the injection site (e.g., viral or bacterial infections, metastatic cells). In addition, the release of growth factors by platelets in the vicinity of a local tumour may facilitate its expansion. Also, infectious and oncologic diseases have the potential to alter the platelet phenotype and secretome toward an inflammatory state, which could lead to local inflammation [36, 38]. There is no compelling scientific evidence based on clinical studies to support the safety of PRP in these conditions. Consequently, the precautionary principle dictates that, in the presence of such pathology, priority should be given to alternative treatments if the risk is lower and the efficacy is comparable.

**Overarching principle 4: Patients must receive clear, personalised information about the potential benefits and risks of PRP injection, taking into account the medical history and comorbidities, and the patient’s informed consent must be recorded in the medical file.**

Grade D

**Appropriate with strong agreement**

Number of ratings: 29

Median = 9; range = 9–9

It is the physician's responsibility to provide the patient with the most accurate and detailed information possible about the proposed treatment plan. This information should include a comprehensive overview of the expected benefits and potential risks, taking into account the patient's individual clinical characteristics. From a medical and legal perspective, patient consent must be obtained and documented in the patient's medical record [40, 51]

**Recommendations for infectious diseases (Table 2)**

**Recommendation 1: In patients with HIV infection, PRP injection may be performed if the viral load is undetectable and the CD4 count is >350/mm^3^.**

Grade C

**Appropriate with strong agreement**

Number of ratings: 20

Median = 8; range = 7–9

Human Immunodeficiency Virus (HIV) infection can be associated with specific inflammatory rheumatologic disorders, although the underlying mechanism remains uncertain. The presence of HIV has been identified in the synovial fluid of patients with arthritis, which could indicate a potential pathophysiologic role for the virus [1, 31]. In addition, HIV is present in platelets both when the viral load is positive and when it is negative but the immune response is inadequate, as indicated by a CD4 count < 350/mm³ [45]. In this case, HIV infection was found associated with platelet activation, characterised by a pro-inflammatory secretory profile, which may be detrimental [5, 53]. Moreover, optimal antiviral treatment must be prioritized in cases in which the viral load remains positive. Although this issue concerns the physician rather than the patient, there is potential risk of accidental blood exposure in case of a positive viral load during the preparation and injection of PRP.

In light of the aforementioned considerations, experts agree that PRP injection should be limited to treated HIV-positive patients with virologic and immunologic responsiveness. No specific timeframe was outlined for the last viral load and CD4 count. However, the most recent test conducted by the infectiologist as part of the standard monitoring protocol (usually every 6 months) will be sufficient, except when the patient has recently initiated or modified their antiviral treatment or if there have been irregularities in the follow-up.

**Recommendation 2: In patients with hepatitis B virus infection, PRP injection may be performed if the viral load is undetectable.**

Grade D

**Appropriate with strong agreement**

Number of ratings: 20

Median = 8.5; range = 7–9

Hepatitis B virus (HBV) has been identified in the synovial membrane, particularly in individuals with rheumatoid arthritis. This observation has led to the hypothesis that HBV may play a pathogenic role in this disease [13]. Furthermore, HBV can penetrate into platelets, leading to their activation, which could be locally deleterious. Finally, there is a potential risk of accidental blood exposure for the physician in cases of a positive viral load during the preparation and injection of PRP.

Consequently, experts concur that PRP injection should be restricted to HBV-positive patients with a negative viral load. No specific timeframe was defined for the last viral load. Nevertheless, the most recent test conducted by the infectiologist as part of the standard monitoring protocol (usually every 3-6 months) will be sufficient, except in cases in which the patient has recently initiated or modified their antiviral treatment or if there have been irregularities in the follow-up.

**Recommendation 3: In patients with hepatitis C virus infection, PRP injection should be performed only after antiviral treatment has been completed.**

Grade D

**Appropriate with strong agreement**

Number of ratings: 20

Median = 9; range = 7–9

Hepatitis C virus (HCV) is present in platelets, leading to their activation [3], which could be locally deleterious. HCV plays a role in the pathogenesis of certain systemic diseases affecting the joints, such as cryoglobulinemia. However, HCV does not appear to have tropism for synovium and/or synovial cells [15, 43]. As with HIV and HBV, there is a potential risk of accidental blood exposure for the physician in the event of a positive viral load during the preparation and injection of PRP.

Currently, HCV infection is treated systematically because the treatment is highly effective and has a duration of only 12 weeks. As with HIV and HBV, the priority should be the administration of antiviral treatment; however, for HCV, the PRP injection should be delayed until the end of the treatment, given the relatively brief duration of the latter. After successful antiviral treatment (negative viral load), there is no requirement for further viral load assessment. Consequently, PRP injection can be performed without the need for systematic viral load monitoring.

**Recommendation 4: In patients with an acute viral infectious syndrome, PRP injection may be performed if the patient has no general symptoms (fever, chills, etc.) or signs of bacterial infection and if the viral syndrome is improving.**

Grade D

**Appropriate with strong agreement**

Number of ratings: 20

Median = 8; range = 7–9

Acute viral infectious syndrome is a common clinical situation in patients who are scheduled to receive an injection for MSK condition. The physician's approach to this situation is heterogeneous, regardless of the type of product to be injected. Experts considered that systematically postponing the injection is probably excessive, especially in the case of viral infection with no severity criteria. Because the main risk is secondary bacterial infection, which could lead to local inoculation of germs via the PRP, experts insist on the assessment of general symptoms (e.g., fever and chills) or other signs suggestive of bacterial infection (e.g., sinusitis or pulmonary infection complicating an ear, nose, and throat [ENT] viral infection), and symptom evolution. They suggested that PRP could be performed in the absence of general symptoms and signs of bacterial infection and when the patient describes a progressive alleviation of symptoms. Conversely, a patient with an incipient viral syndrome should not receive an injection, even with no signs of severity, because we do not know how the syndrome will develop. Experts acknowledged that a viral translocation within the joint cannot be excluded, given that certain viruses, such as parvovirus B19, have an articular tropism and have been identified in joint tissues, especially the synovial membrane [11]. However, no data are available for the most common viruses causing ENT infections, such as rhinovirus. Consequently, PRP injection should also be avoided when articular symptoms are associated with the viral syndrome because they may indicate a tropism and a deleterious effect of the virus on joint tissues.

Finally, experts insist that the precautionary principle should be followed when there is the slightest doubt. Of note, this recommendation was the subject of much debate and less consensus, with some experts arguing that PRP injection is never an emergency and that waiting until the viral syndrome has completely healed is reasonable.

**Recommendation 5: In patients with a bacterial infection requiring antibiotic therapy of less than 3 months, PRP injection may only be performed once the treatment has been completed.**

Grade C

**Appropriate with strong agreement**

Number of ratings: 20

Median = 9; range = 8–9

The contraindication to PRP injection in cases of bacterial infection is justified by the risk of bacteremia [14, 50] and therefore translocation of bacteria to the injection site via plasma or, more rarely, directly via platelets [53]. For most bacterial infections, the duration of antibiotic treatment, although variable, is often a few days to a few weeks, and much more rarely 2-3 months, particularly with material infections (e.g., prostheses) or certain forms of endocarditis. Consequently, experts agree that PRP injection can reasonably be postponed until the end of antibiotic treatment in cases of bacterial infections that warrant treatment for less than 3 months.

**Recommendation 6: In patients with a controlled bacterial infection requiring antibiotic therapy for more than 3 months, PRP injection may be performed only after infectiologist agreement.**

Grade D

**Appropriate with strong agreement**

Number of ratings: 20

Median = 9; range = 8–9

Only a few bacterial infections, mostly with a "chronic" evolution, can justify prolonged antibiotic treatment for more than 3 months . This is the case, for example, with tuberculosis [26] and some other mycobacterial infections [27] and Whipple's disease [9]. In these specific contexts, experts considered that postponing PRP injection for several months can be excessive in a patient with pain and severe functional limitations due to MSK disease and in whom infection seems controlled. Therefore, if the infectiologist confirms that the infection is well controlled and there is no risk of bacterial translocation via the PRP injection, the PRP injection may possibly be performed before the end of the antibiotic treatment. Finally, certain antibiotics have the potential to induce thrombocytopenia [4]. Therefore, a platelet count must be performed immediately before PRP injection in such patients.

**Recommendation 7: In patients with chronic renal failure requiring dialysis, PRP injection may be considered, but the patient should be carefully monitored for signs of bacteremia and the injection must be avoided on the day of dialysis.**

Grade C

**Appropriate with relative agreement**

Number of ratings: 20

Median = 8; range = 5–9

Patients requiring dialysis for chronic renal failure are at high risk of bacteremia [18, 47] due to the recurrent dialysis but also because they are often immunocompromised owing to their initial pathology and often have severe associated comorbidities. However, these patients may be on dialysis during several years, and not offering them local treatment for their MSK pathology after failure of painkillers/physiotherapy seems to be excessive, especially knowing that surgery is even more risky. Therefore, PRP injection could reasonably be allowed but with maximum precautions, in particular by carefully assessing signs of bacteremia.

The risk of bacteremia is highest during and immediately after dialysis. At the same time, if PRP is injected just before dialysis, there is a risk of obtaining poor-quality PRP because it contains many accumulated molecules that have not been purified by the kidney and whose harmful effects are questionable. Hence, not performing PRP injection on the day of dialysis (either before or after) seems logical. This recommendation was the subject of less broad consensus, with a small minority of experts considering that the risk associated with PRP injection in this context exceeds the expected benefit, because of the infectious risk described above or because of excessive platelet activation associated with the renal disease at the origin of a potentially harmful inflammatory profile [28].

**Recommendation 8: In patients with prolonged and stabilized prescription of immunosuppressive agents, PRP injection may be performed, but particular attention must be paid to the presence of concomitant infection.**

Grade C

**Appropriate with strong agreement**

Number of ratings: 20

Median = 8.5; range = 7–9

Immunosuppressive drugs increase the risk of all infections [6, 24]. However, given the often long duration of this treatment, experts considered that it was excessive to contraindicate PRP injection for patients with a MSK pathology, after failure of analgesics, physiotherapy and possibly other injectable treatments. For this reason, PRP injection could be performed in these patients but on the condition that a particular attention is paid to the presence of concomitant infection. Taking into account the concerns of some experts, we have added the notion of prolonged use of the immunosuppressant, considering that if the treatment is transient, as in the case of hematologic transplant, waiting until the end of treatment is recommended. Also, treatment stability was considered necessary to ensure good tolerance without recurrent infections, especially because doses may be higher in the initial phase of treatment.

**Recommendation 9: In patients with dental infection or invasive oral procedures, PRP injection should not be performed until the completion of treatment and healing.**

Grade C

**Appropriate with strong agreement**

Number of ratings: 20

Median = 9; range = 9–9

Dental infections have a bacterial etiology and must be treated with the same precautions as for any other bacterial infection, particularly given their propensity to cause bacteremia due to the translocation of oral bacteria into the bloodstream [48]. Therefore, PRP injection is inadvisable in cases of dental infection. Similarly, the risk of bacteremia (usually transient) is high in invasive oral procedures [39], so PRP injection before the end of the treatment and complete healing seems unreasonable.

**Recommendation 10: In asymptomatic patients with no known infectious pathology, no additional tests for infection are needed.**

Grade D

**Appropriate with strong agreement**

Number of ratings: 20

Median = 9; range = 9–9

The objective of this recommendation was to obviate the need for an exhaustive and costly infectious check-up before PRP injection in all patients. In the absence of symptoms or previous infectious disease, there is no rationale for exhaustive investigations. Also, searching for HIV, HBV or HCV infection was considered excessive in all patients in the absence of clinical elements. Conversely, in case of a known infectious pathology, particularly a chronic viral infection (HIV, HBV, HCV), recent biologic tests are mandatory (recommendations 5-7). Moreover, in the presence of infectious symptoms, PRP injection is usually postponed, except in the case of a viral infection with no severity criteria (recommendation 8), which does not require biologic investigations.

**Recommendations for oncologic diseases (Table 3)**

**Recommendation 11: PRP should not be injected in the vicinity of benign or malignant tumors (bone, synovial or soft tissue) or metaplasia (such as primary synovial chondromatosis).**

Grade D

**Appropriate with strong agreement**

Number of ratings: 21

Median = 9; range = 8–9

The activated platelets present in the injected PRP release numerous growth factors known to stimulate cell proliferation and/or local vascularisation [2, 36, 37]. Consequently, there is a theoretical risk of local proliferation of tumor or metaplastic tissues. Hence, experts considered that PRP should not be injected in the vicinity of benign or malignant tumors or metaplasia.

**Recommendation 12: In patients with solid cancers undergoing diagnosis or considered active, PRP injection should not be performed, except in exceptional situations to be discussed with the oncologist.**

Grade D

**Appropriate with strong agreement**

Number of ratings: 21

Median = 9; range = 9–9

During the diagnosis or treatment of a solid cancer, its evolution and the risk of tumour dissemination remain uncertain. One of the mechanisms by which tumour dissemination occurs is via the plasma passage of tumour cells, which are also called micrometastases. Direct and indirect crosstalk between activated platelets and tumour cells could also promote metastasis [36]. Therefore, the possibility of tumour cells being present in PRP and then seeded at the PRP injection site cannot be discounted, and although rare, the joint may be the site of a metastasis [33]. Furthermore, the platelet phenotype undergoes alterations in the presence of cancer [36], which results in a pro-inflammatory profile that could have a detrimental effect on the tendon or joint. Consequently, in these clinical contexts, PRP injection is contraindicated. However, the experts considered that there are exceptional situations in which PRP injection could be discussed in a patient with active cancer, such as in palliative care. In these situations, the oncologist's consent is always required.

**Recommendation 13: In patients with non-metastatic solid cancer considered in remission by the oncologist after the end of treatment, PRP injection may be performed.**

Grade C

**Appropriate with strong agreement**

Number of ratings: 21

Median = 9; range = 7–9

The period of time that must elapse before a cancer is considered cured varies depending on the type of cancer. Some cancers, such as breast cancer or melanoma, may recur several years after the initial diagnosis. Therefore, defining a fixed cure time for all cancers is somewhat artificial. Taking into account remission confirmed by the oncologist was deemed more appropriate. The concept of remission may be subject to variation in accordance with the initial extent of the cancer. Of note, non-metastatic cancers, which can be localised or locally advanced (i.e., with lymph-node involvement in the drainage area of the tumour), are considered in remission after completion of treatment if there is no longer any tumour tissue present, which is in contrast to remission in metastatic cancers, discussed in detail in recommendation 14. Accordingly, most oncology experts (with one exception) considered PRP injection reasonable in terms of the benefit–risk ratio in these patients without waiting for the longer “cure” period. As reassuring data, 2 studies from the same center examined injection of autologous PRP into the sentinel lymph node scar after biopsy or into the subcutaneous venous access after treatment completion in 163 and 89 patients with breast cancer and did not find tumour development in the injection zone [20, 21].

The recommendation specifies that the cancer treatment must be completed. Hormone therapy is an exception because it is usually given for several years after remission. Therefore, the recommendation allows PRP injection as part of hormone therapy if the cancer is considered in remission. The experts emphasized the need for absolute certainty regarding the patient's remission status, which must be explicitly stated in the latest letter from the oncologist and not merely asserted by the patient. If there is any doubt, the oncologist should be consulted.

**Recommendation 14: In patients with metastatic solid cancer under treatment or not and considered in remission, PRP injection may be performed only after oncologist agreement.**

Grade D

**Appropriate with strong agreement**

Number of ratings: 21

Median = 9; range = 8–9

In the context of metastatic cancers, the concept of remission is more complex. It can be defined as the absence of any residual tumour or, alternatively, as the control of the cancer without the complete eradication of the tumour mass. In this situation, the opinion of the oncologist is essential to assess the risk of tumour cell dissemination due to PRP injection, which depends on each cancer situation. The continuation of oncologic treatment or not does not alter this approach because it may be continued for a long time while the patient with metastasis is considered to be in remission (e.g., in the case of immunotherapy).

**Recommendation 15: In patients with a history of solid cancer that the oncologist considers cured, PRP injection may be performed.**

Grade D

**Appropriate with strong agreement**

Number of ratings: 21

Median = 9; range = 7–9

If the cancer is considered cured by the oncologist, there are no contraindications to PRP injection. As for the "remission" status (recommendation 13), there must be no doubt about the "cured" status of the cancer. If there is any doubt on this point, the oncologist should be consulted for confirmation. Of note, one of the 4 oncologic experts suggested the inclusion of a written document from the oncologist as a prerequisite for PRP injection. However, enforcing this requirement may be difficult if the cancer was treated several years previous. In addition, the recommendation does not specify a timeframe for the cancer to be cured. A 5-year period is often suggested but may vary depending on the type of solid cancer.

**Recommendations for hematologic diseases (Table 4)**

**Recommendation 16: A significant abnormality in the blood count should be investigated before PRP injection.**

Grade D

**Appropriate with strong agreement**

Number of ratings: 22

Median = 9; range = 9–9

If an abnormality in blood count with unknown origin is detected and there is no obvious explanation in the patient's history, the abnormality should be investigated before PRP injection which is never an emergency. In addition, the discovery of a hematologic disease may constitute a contraindication to PRP injection (recommendations 18-22) and, consequently, any injection should be avoided until a final diagnosis. Experts recognised that the term “significant abnormalities” is vague, but proposing a threshold for each blood line seems impossible. This situation remains at the discretion of the clinician, who must investigate this anomaly as they would independently of the context of PRP.

**Recommendation 17: Previously investigated thrombocytopenia > 50,000/mm3, excluding hematologic malignancies, is not a contraindication to PRP injection.**

Grade D

**Appropriate with strong agreement**

Number of ratings: 22

Median = 8; range = 7–9

The objective of these recommendations is to assess the risk of PRP adverse effects, not its reduced efficacy. Therefore, there was no risk of specific adverse effects linked to thrombocytopenia as long as the platelet count was > 50,000/mm^3^ (with risk of bleeding). The only situation distinguished here is the existence of a malignant hemopathy because thrombocytopenia may reflect the persistence of active disease and may be accompanied by substantial platelet function abnormalities (see subsequent recommendations).

**Recommendation 18: In patients with a hematologic malignancy undergoing diagnosis, or not considered stabilized, PRP injection should not be performed, except in exceptional situations to be discussed with the hematologist.**

Grade D

**Appropriate with strong agreement**

Number of ratings: 22

Median = 9; range = 8–9

As for a solid cancer (recommendation 12), not performing PRP injection in a patient with a hemopathy that is in the process of being diagnosed or not stabilized seems reasonable. Indeed, even if the hematologic malignancy is not associated with risk of "local inoculation" of neoplastic cells that could lead to metastasis at the injection site after PRP injection, it can lead to quantitative changes but also dysfunction of platelets that can cause local adverse effects, especially because of their inflammatory phenotype.

**Recommendation 19: In patients with a hematologic malignancy considered in remission by the hematologist after the completion of treatment (including post-transplant immunosuppressive therapy), PRP injection may be performed if there are no platelet count abnormalities.**

Grade D

**Appropriate with strong agreement**

Number of ratings: 22

Median = 9; range = 7–9

As previously described (recommendation 18), the risk is mainly related to the change in platelet phenotype associated with the hemopathy, characterised particularly by a pro-inflammatory profile. PRP injection may be performed after achieving remission of the hemopathy at the end of treatment and in the absence of abnormalities in the platelet line. A normal platelet count is required because quantitative abnormalities may reflect the persistence of active disease and may be associated with significant risk of platelet dysfunction [42], the latter being more difficult to assess in clinical practice. Treatment completion includes immunosuppressive drugs prescribed after stem cell transplantation because they induce a high risk of infection in these already frail patients and their use is generally transient (a few months).

**Recommendation 20: In patients with stabilized chronic lymphoid hemopathy under treatment or not, PRP injection may be performed if there are no platelet count abnormalities.**

Grade D

**Appropriate with strong agreement**

Number of ratings: 22

Median = 9; range = 8-9

Chronic lymphocytic hemopathies are chronic diseases from which most patients never recover, with a few exceptions such as chronic lymphocytic leukemia after allografts, but which can be stabilised for a long time. Therefore, the question of treating associated pathologies, especially MSK diseases, including PRP injection, will arise more frequently, especially when therapeutic alternatives have failed. Experts decided to distinguish between lymphoid and chronic myeloid hemopathies because hematologists insisted that myeloid hemopathies have a greater impact on platelets, quantitatively but also qualitatively, with the risk of injecting pro-inflammatory platelets [42]. Therefore, allowing PRP injection in stabilised lymphoid hemopathies seems reasonable. The need for a normal platelet count has also been added because an abnormal platelet count may reflect uncontrolled hematologic pathology and may be a potential marker of qualitative abnormalities that are more difficult to assess in clinical practice.

**Recommendation 21: In patients with a stabilized chronic myeloid hemopathy under treatment or not, PRP injection may be performed only after hematologist agreement.**

Grade D

**Appropriate with strong agreement**

Number of ratings: 22

Median = 9; range = 8-9

As mentioned in recommendation 20, hematologist experts insisted that myeloid hemopathies have a greater effect on the platelet lineage (both quantitatively and qualitatively) than other hemopathies, which could significantly affect the cellular composition of PRP. Consequently, the experts recommended that PRP should not be used in patients with chronic myeloid hemopathy without the agreement of the hematologist, even with a normal platelet count.

**Recommendation 22: In patients with monoclonal gammopathy of undetermined significance, PRP injection may be performed.**

Grade D

**Appropriate with relative agreement**

Number of ratings: 22

Median = 9; range = 6–9

By definition, monoclonal gammopathy of undetermined significance (MGUS) corresponds to purely biologic abnormalities with no clinical or biologic signs of end-organ damage. Cell lines are also normal, at least quantitatively, because there may be platelet hyperactivity, particularly with high thrombotic activity [44] but which is not a problem with PRP injection. Therefore, MGUS was not considered a contraindication to PRP injection.

**Recommendation 23: In patients with a hematologic malignancy that the hematologist considers cured, PRP injection may be performed.**

Grade D

**Appropriate with strong agreement**

Number of ratings: 22

Median = 9; range = 8–9

As for solid tumours, all experts agreed that a previous hematologic pathology, declared cured by the hematologist, does not contraindicate PRP injection. There must be no doubt as to the "cured" status of the hemopathy, and if there is any doubt, the hematologist should be contacted for confirmation.

**References:**

1. Adizie T, Moots RJ, Hodkinson B, French N, Adebajo AO (2016) Inflammatory arthritis in HIV positive patients: A practical guide. BMC Infect Dis 16:100

2. Andrade SS, Sumikawa JT, Castro ED, Batista FP, Paredes-Gamero E, Oliveira LC, Guerra IM, Peres GB, Cavalheiro RP, Juliano L, Nazário AP, Facina G, Tsai SM, Oliva MLV, Girão MJBC (2017) Interface between breast cancer cells and the tumor microenvironment using platelet-rich plasma to promote tumor angiogenesis - influence of platelets and fibrin bundles on the behavior of breast tumor cells. Oncotarget 8(10):16851–16874

3. Ariede JR, Pardini MI de MC, Silva GF, Grotto RMT (2015) Platelets can be a biological compartment for the Hepatitis C Virus. Braz J Microbiol Publ Braz Soc Microbiol 46(2):627–629

4. Aster RH, Bougie DW (2007) Drug-induced immune thrombocytopenia. N Engl J Med 357(6):580–587

5. Awamura T, Nakasone ES, Gangcuangco LM, Subia NT, Bali A-J, Chow DC, Shikuma CM, Park J (2023) Platelet and HIV Interactions and Their Contribution to Non-AIDS Comorbidities. Biomolecules 13(11):1608

6. Baddley JW, Cantini F, Goletti D, Gómez-Reino JJ, Mylonakis E, San-Juan R, Fernández-Ruiz M, Torre-Cisneros J (2018) ESCMID Study Group for Infections in Compromised Hosts (ESGICH) Consensus Document on the safety of targeted and biological therapies: an infectious diseases perspective (Soluble immune effector molecules [I]: anti-tumor necrosis factor-α agents). Clin Microbiol Infect Off Publ Eur Soc Clin Microbiol Infect Dis 24 Suppl 2:S10–S20

7. Beaufils P, Dejour D, Filardo G, Monllau JC, Menetrey J, Seil R, Becker R (2023) ESSKA consensus initiative: why, when and how? J Exp Orthop 10(1):101

8. Beaufils P, Saffarini M, Karlsson J, Hirschmann MT, Prill R, Becker R, Hantes M, Monllau JC (2024) High scientific value of consensus is based on appropriate and rigorous methodology: The ESSKA formal consensus methodology. Knee Surg Sports Traumatol Arthrosc Off J ESSKADOI: 10.1002/ksa.12390

9. Boumaza A, Ben Azzouz E, Arrindell J, Lepidi H, Mezouar S, Desnues B (2022) Whipple’s disease and Tropheryma whipplei infections: from bench to bedside. Lancet Infect Dis 22(10):e280–e291

10. Burns PB, Rohrich RJ, Chung KC (2011) The levels of evidence and their role in evidence-based medicine. Plast Reconstr Surg 128(1):305–310

11. Cassinotti P, Siegl G, Michel BA, Brühlmann P (1998) Presence and significance of human parvovirus B19 DNA in synovial membranes and bone marrow from patients with arthritis of unknown origin. J Med Virol 56(3):199–204

12. Chang K-V, Hung C-Y, Aliwarga F, Wang T-G, Han D-S, Chen W-S (2014) Comparative effectiveness of platelet-rich plasma injections for treating knee joint cartilage degenerative pathology: a systematic review and meta-analysis. Arch Phys Med Rehabil 95(3):562–575

13. Chen Y-L, Lin J-Z, Mo Y-Q, Ma J-D, Li Q-H, Wang X-Y, Yang Z-H, Yan T, Zheng D-H, Dai L (2018) Deleterious role of hepatitis B virus infection in therapeutic response among patients with rheumatoid arthritis in a clinical practice setting: a case-control study. Arthritis Res Ther 20(1):81

14. Cillóniz C, Gabarrús A, Almirall J, Amaro R, Rinaudo M, Travierso C, Niederman M, Torres A (2016) Bacteraemia in outpatients with community-acquired pneumonia. Eur Respir J 47(2):654–657

15. Cimmino MA, Picciotto A, Sinelli N, Brizzolara R, Accardo S (1997) Has hepatitis C virus a specific tropism for the synovial membrane? Br J Rheumatol 36(4):505–506

16. Cook JL, Purdam CR (2009) Is tendon pathology a continuum? A pathology model to explain the clinical presentation of load-induced tendinopathy. Br J Sports Med 43(6):409–416

17. Coombes BK, Bisset L, Vicenzino B (2010) Efficacy and safety of corticosteroid injections and other injections for management of tendinopathy: a systematic review of randomised controlled trials. Lancet Lond Engl 376(9754):1751–1767

18. Crowe K, White B, Khanna N, Cooke B, Kingsmore DB, Jackson A, Stevenson KS, Kasthuri R, Thomson PC (2021) Epidemiology of bloodstream infections in a Scottish haemodialysis population with focus on vascular access method. J Hosp Infect 110:37–44

19. Dougados M, Betteridge N, Burmester GR, Euller-Ziegler L, Guillemin F, Hirvonen J, Lloyd J, Ozen S, Da Silva J a. P, Emery P, Kalden JR, Kvien T, Matucci-Cerinic M, Smolen J, EULAR (2004) EULAR standardised operating procedures for the elaboration, evaluation, dissemination, and implementation of recommendations endorsed by the EULAR standing committees. Ann Rheum Dis 63(9):1172–1176

20. Eichler C, Baucks C, Üner J, Pahmeyer C, Ratiu D, Gruettner B, Malter W, Warm M (2020) Platelet-Rich Plasma (PRP) in Breast Cancer Patients: An Application Analysis of 163 Sentinel Lymph Node Biopsies. BioMed Res Int 2020:3432987

21. Eichler C, Üner J, Thangarajah F, Radosa J, Zinser M, Fischer LA, Puppe J, Warm M, Malter W, Lenz C (2022) Platelet-rich plasma (PRP) in oncological patients: long-term oncological outcome analysis of the treatment of subcutaneous venous access device scars in 89 breast cancer patients. Arch Gynecol Obstet 306(4):1171–1176

22. Eymard F, Ornetti P, Maillet J, Noel É, Adam P, Legré-Boyer V, Boyer T, Allali F, Gremeaux V, Kaux J-F, Louati K, Lamontagne M, Michel F, Richette P, Bard H, GRIP (Groupe de Recherche sur les Injections de PRP, PRP Injection Research Group) (2021) Intra-articular injections of platelet-rich plasma in symptomatic knee osteoarthritis: a consensus statement from French-speaking experts. Knee Surg Sports Traumatol Arthrosc Off J ESSKA 29(10):3195–3210

23. Filardo G, Previtali D, Napoli F, Candrian C, Zaffagnini S, Grassi A (2021) PRP Injections for the Treatment of Knee Osteoarthritis: A Meta-Analysis of Randomized Controlled Trials. Cartilage 13(1_suppl):364S-375S

24. Fishman JA (2007) Infection in solid-organ transplant recipients. N Engl J Med 357(25):2601–2614

25. Fitzpatrick J, Bulsara MK, McCrory PR, Richardson MD, Zheng MH (2017) Analysis of Platelet-Rich Plasma Extraction: Variations in Platelet and Blood Components Between 4 Common Commercial Kits. Orthop J Sports Med 5(1):2325967116675272

26. Furin J, Cox H, Pai M (2019) Tuberculosis. Lancet Lond Engl 393(10181):1642–1656

27. Gardini G, Gregori N, Matteelli A, Castelli F (2022) Mycobacterial skin infection. Curr Opin Infect Dis 35(2):79–87

28. Gomchok D, Ge R-L, Wuren T (2023) Platelets in Renal Disease. Int J Mol Sci 24(19):14724

29. Haute Autorité de Santé (2015) Development of good practice guidelines: “Formal consensus" method. Methodological guide. Available at: https://pprod-web.has-sante.fr/upload/docs/application/pdf/2018-03/good_practice_guidelines_fc_method.pdf

30. Hohmann E, Tetsworth K, Glatt V (2021) Platelet-Rich Plasma Versus Corticosteroids for the Treatment of Plantar Fasciitis: A Systematic Review and Meta-analysis. Am J Sports Med 49(5):1381–1393

31. Hughes RA, Macatonia SE, Rowe IF, Keat AC, Knight SC (1990) The detection of human immunodeficiency virus DNA in dendritic cells from the joints of patients with aseptic arthritis. Br J Rheumatol 29(3):166–170

32. Irby A, Gutierrez J, Chamberlin C, Thomas SJ, Rosen AB (2020) Clinical management of tendinopathy: A systematic review of systematic reviews evaluating the effectiveness of tendinopathy treatments. Scand J Med Sci Sports 30(10):1810–1826

33. Khurram R, Khurram A, Chaudhary K (2020) Index case of synovial metastasis in a patient with transitional cell carcinoma of the bladder. BMJ Case Rep 13(6):e235084

34. Laver L, Filardo G, Sanchez M, Magalon J, Tischer T, Abat F, Bastos R, Cugat R, Iosifidis M, Kocaoglu B, Kon E, Marinescu R, Ostojic M, Beaufils P, de Girolamo L, ESSKA‐ORBIT Group (2024) The use of injectable orthobiologics for knee osteoarthritis: A European ESSKA-ORBIT consensus. Part 1-Blood-derived products (platelet-rich plasma). Knee Surg Sports Traumatol Arthrosc Off J ESSKA 32(4):783–797

35. Li AK, Stavrakis AI, Photopoulos C (2022) Platelet-rich plasma use for hip and knee osteoarthritis in the United States. The Knee 39:239–246

36. Li S, Lu Z, Wu S, Chu T, Li B, Qi F, Zhao Y, Nie G (2024) The dynamic role of platelets in cancer progression and their therapeutic implications. Nat Rev Cancer 24(1):72–87

37. Luzo ACM, Fávaro WJ, Seabra AB, Durán N (2020) What is the potential use of platelet-rich-plasma (PRP) in cancer treatment? A mini review. Heliyon 6(3):e03660

38. Mandel J, Casari M, Stepanyan M, Martyanov A, Deppermann C (2022) Beyond Hemostasis: Platelet Innate Immune Interactions and Thromboinflammation. Int J Mol Sci 23(7):3868

39. Martins CC, Lockhart PB, Firmino RT, Kilmartin C, Cahill TJ, Dayer M, Occhi-Alexandre IGP, Lai H, Ge L, Thornhill MH (2024) Bacteremia following different oral procedures: Systematic review and meta-analysis. Oral Dis 30(3):846–854

40. Maugars Y, Bard H, Latourte A, Senbel É, Flipo R-M, Eymard F, a French group multidisciplinary experts in interventional rheumatology (2023) Musculoskeletal corticosteroid injections: Recommendations of the French Society for Rheumatology (SFR). Joint Bone Spine 90(2):105515

41. Mobasheri A, Loeser R (2024) Clinical phenotypes, molecular endotypes and theratypes in OA therapeutic development. Nat Rev Rheumatol 20(9):525–526

42. Moscardó A, Latorre A, Santos MT, Bonanad S, Vallés J (2015) Platelet function in malignant hematological disorders. Curr Opin Oncol 27(6):522–531

43. Nadeem A-ED, Thomas P, Ulf M-L, Elena N, Anggakusuma A, Mohamed BM, Frank P, Patrick B (2015) Cell culture-derived HCV cannot infect synovial fibroblasts. Sci Rep 5:18043

44. O’Sullivan LR, Meade-Murphy G, Gilligan OM, Mykytiv V, Young PW, Cahill MR (2021) Platelet hyperactivation in multiple myeloma is also evident in patients with premalignant monoclonal gammopathy of undetermined significance. Br J Haematol 192(2):322–332

45. Real F, Capron C, Sennepin A, Arrigucci R, Zhu A, Sannier G, Zheng J, Xu L, Massé J-M, Greffe S, Cazabat M, Donoso M, Delobel P, Izopet J, Eugenin E, Gennaro ML, Rouveix E, Cramer Bordé E, Bomsel M (2020) Platelets from HIV-infected individuals on antiretroviral drug therapy with poor CD4+ T cell recovery can harbor replication-competent HIV despite viral suppression. Sci Transl Med 12(535):eaat6263

46. Scherlinger M, Richez C, Tsokos GC, Boilard E, Blanco P (2023) The role of platelets in immune-mediated inflammatory diseases. Nat Rev Immunol 23(8):495–510

47. Skov Dalgaard L, Nørgaard M, Jespersen B, Jensen-Fangel S, Østergaard LJ, Schønheyder HC, Søgaard OS (2015) Risk and Prognosis of Bloodstream Infections among Patients on Chronic Hemodialysis: A Population-Based Cohort Study. PloS One 10(4):e0124547

48. Šutej I, Peroš K, Trkulja V, Rudež I, Barić D, Alajbeg I, Pintarić H, Stevanović R, Lepur D (2020) The epidemiological and clinical features of odontogenic infective endocarditis. Eur J Clin Microbiol Infect Dis Off Publ Eur Soc Clin Microbiol 39(4):637–645

49. Testa G, Giardina SMC, Culmone A, Vescio A, Turchetta M, Cannavò S, Pavone V (2021) Intra-Articular Injections in Knee Osteoarthritis: A Review of Literature. J Funct Morphol Kinesiol 6(1):15

50. Thornton HV, Hammond A, Hay AD (2018) Urosepsis: a growing and preventable problem? Br J Gen Pract J R Coll Gen Pract 68(675):493–494

51. Uson J, Rodriguez-García SC, Castellanos-Moreira R, O’Neill TW, Doherty M, Boesen M, Pandit H, Möller Parera I, Vardanyan V, Terslev L, Kampen WU, D’Agostino M-A, Berenbaum F, Nikiphorou E, Pitsillidou IA, de la Torre-Aboki J, Carmona L, Naredo E (2021) EULAR recommendations for intra-articular therapies. Ann Rheum Dis 80(10):1299–1305

52. Vicenzino B, de Vos R-J, Alfredson H, Bahr R, Cook JL, Coombes BK, Fu SN, Gravare Silbernagel K, Grimaldi A, Lewis JS, Maffulli N, Magnusson SP, Malliaras P, Mc Auliffe S, Oei EHG, Purdam C, Rees JD, Rio EK, Scott A, Speed C, Akker-Scheek I van den, Weir A, Wolf JM, Zwerver J (2020) ICON 2019-International Scientific Tendinopathy Symposium Consensus: There are nine core health-related domains for tendinopathy (CORE DOMAINS): Delphi study of healthcare professionals and patients. Br J Sports Med 54(8):444–451

53. Youssefian T, Drouin A, Massé J-M, Guichard J, Cramer EM (2002) Host defense role of platelets: engulfment of HIV and Staphylococcus aureus occurs in a specific subcellular compartment and is enhanced by platelet activation. Blood 99(11):4021–4029
